# Salivary Progesterone Levels in Female Patients with a History of Idiopathic Scoliosis: A Retrospective Cross-Sectional Study

**DOI:** 10.3390/clinpract12030038

**Published:** 2022-05-11

**Authors:** Mark W. Morningstar, Megan N. Strauchman

**Affiliations:** Natural Wellness & Pain Relief Center, Grand Blanc, MI 48423, USA; strawk_man@yahoo.com

**Keywords:** hormone, musculoskeletal disease, orthopedic disorders, progesterone, scoliosis

## Abstract

Idiopathic scoliosis is a condition characterized by a three-dimensional curvature of the spine. However, in addition to the spinal curvature, it has also been reported that patients with idiopathic scoliosis can display other abnormal physiologic parameters, such as hormone imbalances, genetic variants, and micronutrient deficiencies. The present study evaluates the salivary progesterone levels from a historical cohort of patients seeking treatment at a single integrative medicine clinic. A group of female patients with a history of idiopathic scoliosis is compared to a group of non-scoliosis female patients. Salivary progesterone levels were 49% higher in non-scoliosis patients compared to the scoliosis group (*p* < 0.05). When compared by the menstrual cycling status, non-scoliosis females had a 60% higher average progesterone level, while the progesterone level among non-cycling, non-scoliosis females was 39% higher than non-cycling scoliosis females. These results suggest a potential relationship between salivary progesterone and idiopathic scoliosis among female patients.

## 1. Introduction

Idiopathic scoliosis (IS) is a condition characterized by a spinal curvature measuring 10° or more in a radiographic study by Cobb’s angle [1]. It is estimated that 0.47–5.2% of the US population ages 0–17 have this condition, increasing to 8% of the adult population [2]. Conservative treatments for adolescent idiopathic scoliosis are based upon the initial presenting curve magnitude, as well as the growth/maturation status. According to the 2016 SOSORT treatment guidelines [3], curves between 10 and 15 are treated with exercises, while curves between 15 and 50 degrees should be treated with exercises and/or bracing, depending upon the patient’s growth stage. Curves exceeding 50° are typically referred for surgical interventions such as vertebral body tethering or spinal fusion surgery [4]. Most reported treatment options for adults with idiopathic scoliosis are surgical [5]. Adult idiopathic scoliosis patients typically seek treatment due to increased back pain and disability [6].

While these treatment recommendations focus solely on the spinal curvature itself, they do not address some of the non-spinal comorbidities commonly associated with the spinal curvature. These may include vitamin D deficiency [7], neurotransmitter deficiencies and/or signaling abnormalities [8], low bone mineral density [9], and various hormone imbalances [10,11,12]. These discoveries over time have led to more robust hypothetical modeling of scoliosis etiopathogenesis [13].

Investigations into hormonal involvement in idiopathic scoliosis have chiefly focused on estrogen, leptin, and melatonin [13]. Progesterone, by contrast, has not been widely studied within an idiopathic scoliosis etiological model. Progesterone has primarily been studied for its impact on reproductive and neurological function. A study by Kulis et al. showed that serum progesterone levels in girls with idiopathic scoliosis did not differ significantly from controls [14]. However, there is debate about the consistency of serum progesterone values in females throughout a typical menstrual cycle [15]. By contrast, normative salivary progesterone levels throughout a normal menstrual cycle have been previously established [16], including young pre-pubescent females [17], in whom salivary progesterone levels still reach ovulatory levels in most subjects. Salivary progesterone testing has shown consistency across varying laboratories, well-defined reference ranges, and high correlation with mass spectroscopy [18,19].

Given these previous observed hormone differences in idiopathic scoliosis, the present study investigated the occurrence of salivary progesterone differences in scoliosis patients. The current study reports on the test results of patients with and without idiopathic scoliosis and the differences observed in their salivary progesterone levels. Investigations into any relationships between idiopathic scoliosis and salivary progesterone levels have not been previously reported on in the PubMed database.

## 2. Methods and Materials

The data in this study were collected and reported on according to the STROBE format (http://www.strobe-statement.org, accessed on 18 November 2021) for cross-sectional studies. A consecutive sampling of all patient records from a single multidisciplinary medical clinic in Grand Blanc, Michigan, USA, was conducted on patients who sought treatment there for any reason, from January 2018 to November 2020. Patient charts were included in the present study if they met the following inclusion criteria: (1) patient had a history of idiopathic scoliosis, (2) patient was female, and (3) patient completed a salivary hormone test. Using these inclusion criteria, a total of 68 patient files was selected. All patient records meeting the inclusion criteria were included in the sample group. To verify that these patients had a history of adolescent idiopathic scoliosis, the chart needed to show documentation of a scoliosis radiograph taken at the presenting clinic, or that the patient had a scoliosis radiograph performed elsewhere, with a radiology report documenting the existence of idiopathic scoliosis.

**Figure 1:**
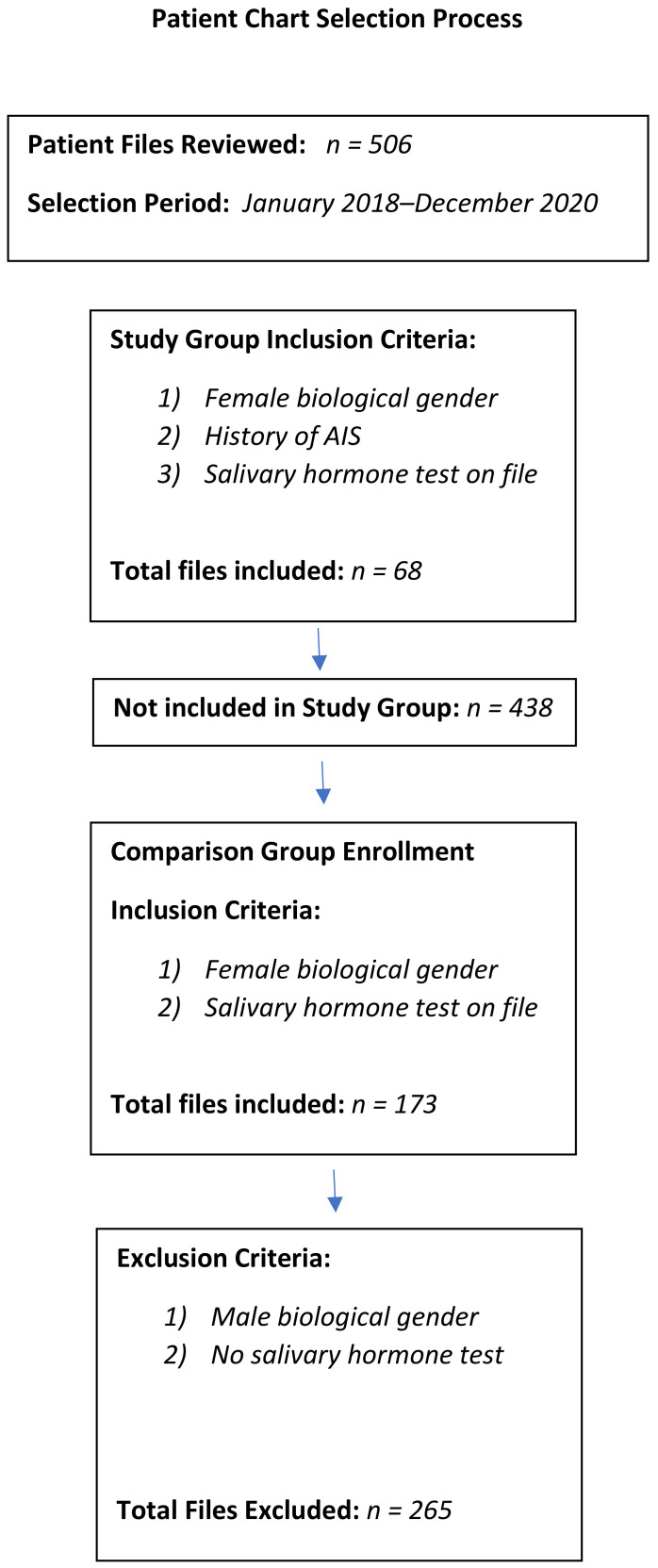
Patient chart selection process.

Once the first group of patient files were collected and grouped, a second data collection was performed from the files not initially selected as part of the study group. For this group, there were 2 inclusion criteria: (1) the patient completed the same salivary hormone test as the first group, and (2) patient was female. Based upon these inclusion criteria, 173 patient files were identified. This collection of files served as the non-scoliosis group. Figure 1 shows a flow chart of the file selection process. Data were only collected from female patients, since female reference progesterone levels are different than male ones. Therefore, males were excluded to improve data homogeneity. It is important to note that both groups were patients at the same clinic, being managed for various symptoms and conditions, not necessarily just scoliosis. For example, a salivary progesterone level may have been ordered for headaches or menstrual symptoms regardless of a patient’s history of scoliosis. None of the patients in either group were pregnant or breastfeeding at the time of salivary collection, due to their potential impacts on hormone ratios. Since oral contraception also affects salivary hormone results, none of the patients whose results were used for this study were on oral contraceptive medication at the time of collection.

The following non-identifying data were extracted from all the files in both groups: age, cycling status, and baseline salivary progesterone level (used when patients had completed more than one salivary hormone test). An advisory review was conducted by the Advarra Institutional Review Board (IRB), where they determined that the study design was exempt from IRB approval.

The average age of the entire scoliosis patient group was 28 years (range 11–62). When subcategorized by menstrual status, the average age of cycling scoliosis patients was 29, and the average non-menarchial age was 26. The average age for the non-scoliosis group was 44 years (range 16–67), 38 years for cycling non-scoliosis patients, and 49 years for non-menarchial, non-scoliosis patients.

The salivary progesterone levels were ordered through Labrix (http://www.doctorsdata.com, accessed on 6 November 2021). Salivary progesterone was chosen for a variety of reasons. Salivary progesterone collection is noninvasive and, hence, easier to obtain from adolescent patients. Normative data for pre-pubescent, pre-menopausal, and post-menopausal females were established [16,20,21], allowing for comparison to the present scoliosis patient group. In a large percentage of healthy subjects, salivary progesterone levels would reach ovulatory levels, even in pre-menarchial females [17]. This may make salivary progesterone a more sensitive marker in pre-menarchial females for comparative purposes. Finally, salivary progesterone levels are reflective of glandular, unbound levels. This contrasts with serum progesterone, which is mainly bound to sex hormone binding globulin and, hence, inactive. Therefore, salivary progesterone levels may provide more clinically meaningful evidence, given that progesterone spikes are detectable in saliva samples, even in pre-pubescent females [17]. All saliva samples collected from patients in the present study were collected between days 19 and 23 of their menstrual cycle if they were normally menstruating, in an effort to collect during their progesterone peak. Post-menopausal and pre-pubescent patients could collect at any time, but were still required to collect all four saliva samples on the same day.

Salivary progesterone levels were compared among the following groups: the scoliosis group and non-scoliosis group. These groups were subdivided according to menstrual status: scoliosis non-menarchial group (pre or post combined), scoliosis menarchial group, non-scoliosis, non-menarchial group, and non-scoliosis menarchial group. The onset of menarche, as well as menopause, is associated with significant changes in hormone production. Therefore, stratification was conducted based upon menstrual status. Student’s *t*-tests were used to compare the average progesterone levels among the stratified subgroups. This was performed to minimize confounding factors, such as cycling status.

## 3. Results

For the scoliosis group, the average salivary progesterone level was 67.79 pg/mL ± 39.08. A total of 50 of the 68 patients were cycling, and 18 were not. Among the 50 cycling scoliosis patients, their average progesterone was 68.42 pg/mL ± 38.15. In the non-menarchial scoliosis patients, their average progesterone was 66.06 pg/mL ± 42.66.

The salivary progesterone for the non-scoliosis group was 101.63 pg/mL ± 51.31. The non-scoliosis patient group had 93 women who were cycling, while 80 were not. The non-scoliosis cycling patients had an average progesterone level of 109.75 pg/mL ± 51.14. The 80 non-scoliosis, non-menarchial patients had average progesterone levels of 91.80 pg/mL ± 50.60. A summary of these data are shown in Table 1, Table 2 and Table 3.

Table 4 shows a summary of the reference range values for normal salivary progesterone values based on cycling status. Intergroup comparisons were determined for each menstrual cycling classification: pre-pubescent, cycling, and post-menopause. The values for both the pre-pubescent scoliosis and non-scoliosis patients were within the normal laboratory reference range, but they were statistically significantly difference from each other. The cycling groups were both lower than the reference range, and were also significantly different from each other (*p* < 0.001). The post-menopause patient groups were statistically the same, and within the normal reference range.

Student’s *t*-tests were then used to determine if any of the observed differences were statistically significant. Table 5 shows a summary of the intragroup *t*-test comparisons and the resultant *p* values. Intragroup comparisons in both the scoliosis and non-scoliosis patient groups showed that differences in menstrual cycling were not statistically significant. These were performed to identify these as confounding variables, and had calculations showing a statistically significant difference. The cycling status did not significantly change the progesterone values in the scoliosis patient group, but was significantly different in the non-scoliosis patient group.

A post hoc power analysis was conducted for the progesterone levels using an online calculator (http://www.clincalc.com, accessed on 7 December 2021). The current sample size for both groups resulted in 100% power to show a 34 pg/mL difference at a 0.05 confidence interval using a continuous endpoint.

## 4. Discussion

The evaluation of two groups of consecutively selected patient charts showed statistically significant differences in the salivary progesterone levels among female patients with a past medical history of adolescent idiopathic scoliosis when compared to a group of patients without a known history of adolescent idiopathic scoliosis. These values were independent of the menstrual cycling status.

It has been known for several decades that scoliosis can continue to progress throughout adulthood after skeletal maturity [22]. Marty-Poumarat et al. [23] showed that adult scoliosis progression is linear and is estimated to occur in 68% of curves measuring ≥ 30° at the time of skeletal maturity, regardless of the curve pattern [4,24]. The proposed theories for this observed progression are primarily biomechanical [5], and have largely ignored the potential for metabolic or endocrine factors to influence curve progression. The present study showed the differences between pre-pubescent and cycling patients with and without idiopathic scoliosis. This added to a growing list of observed differences in metabolic and endocrine factors in those with idiopathic scoliosis. It is at least conceivable that these observed metabolic and endocrine differences may play some role in the continued progression of idiopathic scoliosis throughout the lifespan.

Although it is unknown how progesterone might impact scoliosis onset or progression, progesterone maintains an important influence in the motor memory centers of the brain, such as the hippocampus and the thalamus via synaptic plasticity [25]. Central pattern generator differences have been observed in scoliosis patients [13]. Progesterone’s involvement in central pattern generation would be via the central nervous system upregulation of brain-derived neurotrophic factor (BDNF) receptors [26,27].

There were some limitations involved in this study, based on the study design, which warrants discussion. As with any retrospective design, selection bias was a potential factor. However, as described in the paper, all patient charts within a historical time frame were selected, and both groups of charts comprised all the charts, if the inclusion criteria were fulfilled. However, male patients were excluded from the study. Therefore, the results of the study may not be applicable to male patients with a history of idiopathic scoliosis. Information bias was also addressed by incorporating the menstrual cycling status as an independent variable. We also reported the patient results compared to the reference ranges published by the laboratory. These reference ranges were also based on the menstrual cycling status. The menstrual cycling status was used as an independent variable in efforts to reduce selection bias. Since the outcome measures were obtained from independent laboratory testing not performed by the author and not collected from self-rated surveys or questionnaires, self-selection bias and outcome-based information bias could also be minimized.

One additional limitation was that a certain unknown portion of the non-scoliosis patient group may, in fact, have a previously undiagnosed idiopathic scoliosis. Using the 5–8% estimate of the teen and adult prevalence of idiopathic scoliosis [2], it is possible that up to 13 patients in the non-scoliosis group would be correctly added to the scoliosis group. However, if their progesterone levels were consistent with the scoliosis group, meaning they would be closer to the scoliosis group average versus the non-scoliosis group average, it may have further decreased the resultant *p* value; thus, increasing its statistical significance had they been placed into the corrected group (the scoliosis patient group).

Although the study design did not allow for any assumptions on the potential role of progesterone in scoliosis management, this study did provide the evidence that investigating the role of progesterone in robust scoliosis management in an interventional-type study is warranted.

The results of the present study suggested that salivary progesterone is significantly lower in female patients with a history of idiopathic scoliosis (IS) when compared to female patients with a negative history of IS. The menstrual cycling status did not impact the average levels on intragroup comparison. The study design prevented any extrapolation on causality, nor were these results necessarily applicable to male scoliosis patients. Future investigations are warranted in male patients, as well as comparing salivary and serum progesterone levels in similar groups.

## Figures and Tables

**Table 1:** Progesterone values for entire cohort.

Group	# Patients	Progesterone	# Cycling
Scoliosis	68	67.79 pg/mL	50
Non-Scoliosis	173	101.63 pg/mL	93

Number (#).

**Table 2:** Progesterone values based on pre-menopausal patients (currently cycling).

Cycling Patients	# Patients	Progesterone
Scoliosis	50	68.42 pg/mL
Non-Scoliosis	93	109.75 pg/mL

Number (#).

**Table 3:** Progesterone values for non-cycling patients.

Non-Menarchial Patients	# Patients	Progesterone
Scoliosis	18	66.06 pg/mL
Non-Scoliosis	80	91.80 pg/mL

Number (#).

**Table 4:** Progesterone values compared to laboratory reference ranges.

Patients	Reference Range Progesterone (pg/mL)	Scoliosis Patients (n)	Non-Scoliosis (n)
Pre-Pubescent	<94	46.67 (9) ^1^	86.43 (22)
Cycling	127–446	68.42 (50) ^2^	109.75 (93)
Post-Menopause	18–130	85.44 (9) ^3^	95.53 (58)

^1^ Significant difference at *p* < 0.001. ^2^ Significant difference at *p* < 0.001. ^3^ *p* = 0.609.

**Table 5:** *P* values based on cycling status.

Intragroup Comparison *	Scoliosis Group	Non-Scoliosis Group
Cycling Yes vs. Cycling No	0.827	0.0196 ^ⱡ^

* Student’s *t*-tests, two-sample equal variance, 95% CI. ^ⱡ^ Statistically significant at *p* < 0.05.

## Data Availability

The data presented in this study are available on request from the corresponding author. The data are not publicly available due to privacy reasons.
